# Lens Proteomics Provide Novel Clues for Cataractogenesis: Original Investigation and a Broad Literature Survey

**DOI:** 10.3390/jcm14134737

**Published:** 2025-07-04

**Authors:** Banu Cosar, Mustafa Sehvar Nefesoglu, Meric A. Altinoz, Emel Akgun, Betul Sahin, Ahmet Baykal, Mustafa Serteser

**Affiliations:** 1 Faculty of Medicine, Department of Ophthalmology, Acibadem Mehmet Ali Aydınlar University, Istanbul 34398, Türkiye; 2 Avrupa Vocational School, Kocaeli Health and Technology University, Kocaeli 41001, Türkiye; mustafa.nefesoglu@kocaelisaglik.edu.tr; 3 Ophthalmology Clinic, Atasehir Florence Nightingale Hospital, Istanbul 34750, Türkiye; 4 Faculty of Medicine, Department of Medical Biochemistry, Acibadem Mehmet Ali Aydınlar University, Istanbul 34638, Türkiye; adilmeric.altinoz@saglik.gov.tr (M.A.A.); ahmet.baykal@acibadem.edu.tr (A.B.); mustafa.serteser@acibadem.edu.tr (M.S.); 5 Acıbadem LabMed Clinical Laboratories, Istanbul 34752, Türkiye; emel.akgun@live.acibadem.edu.tr (E.A.); betul.sahin1@bau.edu.tr (B.S.); 6 Department of Biochemistry, Bahcesir University, Istanbul 34349, Türkiye

**Keywords:** cataract, proteomics, visual acuity, dysfunctional lens index

## Abstract

**Background**: Previous proteomic studies provided valuable information about cataracts, but unclarified issues, such as sex and ethnicity-associated differences, remain. This study aimed to provide additional data on cataract-related proteins regarding age, sex, and cataract type. **Methods**: Twenty-six female and seven male Turkish cataract patients were screened for visual acuity and dysfunctional lens index. A nano-LC-MS/MS system and Progenesis QI software v3.0 were used for protein identification and quantification. The remaining data were evaluated with SPSS Version 29.0 software. **Results**: Proteins that showed age-associated changes were mainly involved in cytoskeletal organization. A glyoxalase enzyme, caveolin 1, and HS90B were lower, and RAB8B and ATP6V1B1 were higher in lenses in women. Proteins with lower levels in cataractous lenses than in transparent lenses included filensin and phakinin, concurrent with previous publications, and LCTL, GDI, HSPB1, and EIF4A2, not reported before. Corticonuclear cataracts constituted the only group showing depletions in putatively protective proteins, while the cortical type was the least influenced. ANXA1 and DNHD1 positively, and TCPD, SEC14L2, and PRPS1 proteins negatively correlated with visual acuity. **Conclusions**: This study revealed cataract-related proteins concurrent with earlier studies and new ones hitherto unreported. Despite the low number of patients investigated, the results merit further research, as these new proteins are highly likely to be involved in cataractogenesis.

## 1. Introduction

Cataracts constitute the foremost global cause of blindness, and the risk factors include, but are not limited to, ultraviolet (UV) light, diabetes, steroids, medications or surgeries employed to lower intraocular pressure, uveitis, trauma, and certain occupations [1,2]. Due to low economic status, excess production of biomass cooking fuels, and working in outdoor jobs, cataract rates are higher in less-developed countries. Several epidemiological observations showed that cataracts occur more frequently among women, yet others reported an opposite trend [1]. However, as the smoking likelihood and UV light exposure due to outdoor jobs are more common among men, these factors may blur the epidemiological findings. Hence, there is a higher possibility that women are more vulnerable to cataracts. A meta-analysis investigating 161,947 cataract patients revealed that higher age was significantly correlated with higher prevalence for all cataract types [1]. This finding suggests that, when the lens fiber cells fully differentiate, they cannot recycle proteins, equalizing the lifespan of proteins and cells, and subsequently leading to the intralentricular accumulation of damaged proteins throughout life [3]. Several proteomic studies regarding cataracts provided essential findings. Yet, there is a need for continuing proteomic investigations to understand poorly understood aspects, such as differences in cataract prevalence according to gender or geography. This requirement makes sense, taking into account that the human lens contains over 5000 proteins, which is surprisingly high considering the avascularity and organelle-poor cells within the lens [3]. Another astonishing fact is that, despite its transparency, the lens contains the highest protein percentage among the tissues adjusted to wet weight [4]. Therefore, this research analyzed proteomic differences between transparent and cataractous lenses and age, sex, and cataract type-dependent variations in a Turkish patient cohort. 

## 2. Materials and Methods

### 2.1. Ethics, Design and Ophtalmological Examinations

All patients were informed about the research, and signed informed consent forms were obtained from those who agreed to participate. ATADEK, the ethical committee of Acibadem University (Istanbul, Turkey), provided ethical approval (Decision Number: 2024-18/4; date: 21 November 2024). Exclusion criteria included any corneal, retinal, or optic nerve diseases. Clear and cataractous lenses were obtained during routine phacoemulsification and intraocular lens implantation surgeries, collected in the waste bag of the phacoemulsification machine, transferred to Eppendorf tubes, and stored at −80 °C until analysis. The cohort comprised 33 patients with a median age of 63.5 (range: 45–85), including 26 females and 7 males. The cataracts were localized on the right or left lenses in 17 and 16 patients, respectively. Four samples analyzed with proteomics were transparent lenses, and 29 cataract samples included 4 different types, where the definition of the mixed type represented either cortical + posterior subcapsular, nuclear + posterior subcapsular, or corticonuclear + posterior subcapsular. As Table 1 and Table 2 list other details, they are not repeated here to avoid redundant information. The visual acuities were determined with Snellen charts and then converted to logMar values. The dysfunctional lens index (DLI) is an objective parameter evaluated by the iTrace Visual Function Analyzer (Tracey VFA Visual Function Analyzer, Tracey Technologies, Houston, TX, USA) that quantifies the extent of lens decomposition. DLI values were determined between scores 0 and 10 according to the definitions of the manufacturer’s operator’s manual. In this scoring, lower and higher values indicate dysfunctional and better-functioning lenses, respectively.

### 2.2. Proteomics: LC-MS/MS, Protein Identification and Quantitative Analysis

Lens tissue lysis and homogenization were performed with a UPX solution (Expedion, Expedion Protein Solutions, San Diego, CA, USA) containing a cocktail of protease inhibitors (Thermo Scientific, Waltham, MA, USA). The Bradford protein assay was utilized to measure the protein concentrations, and the filter-aided sample preparation (FASP) method was used to obtain tryptic peptides. Obtaining tryptic peptides was achieved by adding trypsin (Promega, San Luis Obispo, CA, USA) at 1:100 (*w*/*w*) and allowing 18 h to complete the digestion at 37 °C. Before LC-MS/MS analysis, peptide concentrations were quantified with fluorometric peptide assay (Pierce, Appleton, WI, USA), and the final peptide concentration was calibrated to 200 ug/mL using 0.1% formic acid. The nano-LC-MS/MS system (Acquity UPLC M-Class and SYNAPT G2-Si HDMS, Milford, CT, USA) was employed for the tryptic peptide mixture analysis. After loading the peptide mixtures on the trap column (Symmetry C18 5 mm, 180 mm i. d. 20 mm), their separation was achieved through an analytic column (CSH C18, 1.7 mm, 75 mm i. d. 250 mm) with a linear two-h gradient (4–40% Acetonitrile 0.1% (*v*/*v*) FA, 0.300 mL/min flow rate). Glu-1-fibrinopeptide-B (100 fmol/µL) was the lockmass reference at 0.500 mL/min flow rate with 60 s intervals. MS data acquisition was performed with SONAR (a data-independent acquisition mode) with a quadrupole transmission width of 24 Da. Positive ionization at 50–2000 *m*/*z* in the full-scan mode was used to acquire data. Quantitative peptide analysis and protein identification were performed with Progenesis QI for proteomics (v.4.0, Waters, Milford, MA, USA). The statistical package included in Progenesis QI enabled the calculation of expressional changes and *p*-values. Proteins were normalized according to relative quantitation using non-conflicting peptides. The obtained data were filtered by ANOVA according to a *p*-value of 0.01. The proteins with a differential expression level equal to or greater than 1.5-fold change were considered.

### 2.3. Statistical Analysis of Non-Proteomic Data

SPSS statistical software (Version: 29.0, IBM, Armonk, NY, USA) was utilized for statistical analyses. The patients’ features were represented as *n* (percent) and the mean ± standard deviation (SD) for categorical and continuous variables, respectively. A two-tailed Kolmogorov–Smirnov test was employed to evaluate whether the continuous variables exert a Gaussian distribution. The independent-sample *t*-test was used to compare the values of two independent groups. The ANOVA test was performed to compare the values of three or more independent groups. Following these analyses, the post hoc Bonferroni test and Pearson’s correlation analysis were utilized to confirm the statistical significance of intergroup differences and to compare continuous variables, respectively. *p*-values lower than 0.05 were accepted as significant.

## 3. Results

### 3.1. General Features

Table 1 lists the demographic and clinical features of the whole study cohort.

There were 26 female and 7 male cataract patients with a mean age of 62.08 (standard deviation: ±9.32). Seventeen cataracts were localized on the right and sixteen on the left lenses. Four of the investigated lenses were transparent, while five, nine, ten, and five had cortical, nuclear, corticonuclear, and mixed-type cataracts, respectively. The mean ages of patients were 57 ± 2.6, 66.78 ± 2.54, 73.90 ± 1.90, and 63.22 ± 3.81 for the cortical, nuclear, corticonuclear, and mixed-type cataracts groups, respectively.

Table 2 presents data about age, dysfunctional lens index (DLI), and visual acuity (V-CC LogMar) classified according to cataract type or clear lens. The mean visual acuity (V-CC LogMAR) value of the cohort was 0.26 ± 0.25, while the V-CC values were 0.5 ± 0.01, 0.21 ± 0.04, 0.41 ± 0.07, 0.43 ± 0.10, and 0.012 ± 0.001 for the cortical, nuclear, corticonuclear, and mixed-type cataracts groups, respectively. The mean dysfunctional lens index value of the cohort was 5.38 ± 2.76, while the mean DLI values were 7.09 ± 1.43, 6.09 ± 0.82, 3.57 ± 0.85, 4.07 ± 0.76, and 7.51 ± 0.76 3.57 ± 0.85 for the cortical, nuclear, corticonuclear, and mixed-type cataracts groups, respectively.

### 3.2. Protein Differences According to Age

Supplementary Table S1 lists proteins that were significantly correlated with age. A considerably high number of proteins exerted statistical significance in either negative or positive correlations with age.

Among these, 13 proteins were correlated with age with statistical *p*-values less than 0.005 and above 0.001, and 5 proteins were correlated with age with a statistical *p*-value equal to 0.001. Eleven and four proteins were negatively and positively correlated with age at *p*-values lower than 0.001, respectively. This article discusses only the proteins differing with *p*-values less than 0.001. Thus, the reader can refer to the tables to uncover other potentially essential proteins that change during aging. Some proteins correlated with age also demonstrated correlations with visual acuity and dysfunctional lens index parameters or differed between females and males, for which functions were defined in a combined manner without a separate discussion regarding age. In descending order of correlation coefficients, the proteins negatively correlating with age were phakinin (Q13515), Eukaryotic Initiation Factor 4A2 (Q14240), filensin (Q12934), LSAMP (Limbic system-associated membrane protein, Q13449), Lactase-Like protein (Q6UWM7), Catenin β-1 (P35222), Vimentin (P08670), Carbonic anhydrase 14 (Q9ULX7), and ADP Ribosylation Factor 3 (P61204). In descending order of correlation coefficient, the proteins positively correlating with age were Dynein axonemal heavy chain 9 (Q9NYC9), β-crystallin B1 (P53674), Forkhead-associated domain-containing protein 1 (B1AJZ9), and Gamma-crystallin S (P22914).

### 3.3. Protein Differences According to Sex

Supplementary Table S2 lists the mean protein levels classified according to sex. Five proteins with statistically significant expressional variations are shown in bold.

Among these, P32754 (4-hydroxyphenylpyruvate dioxygenase) differed with the highest significance (*p* = 0.003) and existed in about 1.62-fold lower amounts in women’s lenses than in men’s. P21281, Q3135, P50395, and P08238 were the other proteins that displayed sex-related variations in expression. This manuscript provides further information on other protein level variations according to sex. Therefore, they will not be described further here to avoid redundant discussions.

### 3.4. Protein Differences According to Lens Features and Cataract Type

Supplementary Table S3 shows proteins classified according to the lens features (clear vs. cataractous) and cataract types.

Thirty-three proteins had statistically significant expressional changes between different groups, as shown with bold letters, where the last column indicates differences between clean and cataractous lenses and between different cataract types. The *p*-values regarding the expressional differences of Q14240, Q12934, and P04792 proteins were equal to 0.001, while Q13515, Q6UWM7, and P31150 proteins showed expressional variances with *p*-values below 0.001. Only these six proteins having *p* values ≤ 0.001 are discussed in this article. The reader can also focus on the five other varyingly expressed proteins (O76054, Q9ULX7, Q13449, P00387, and P30041) with *p* values ≤ 0.005 that may reveal different aspects relevant to cataract development. The Q13515 (phakinin) protein exhibited reductions in nuclear and corticonuclear cataracts compared with transparent lenses, and its expression in corticonuclear cataracts was approximately 1.53-fold less than that in cortical cataracts. Q6UWM7 (lactase-like protein) was significantly lower in nuclear, corticonuclear, and mixed-type cataracts than in transparent lenses and about 1.15-fold lower in corticonuclear than in cortical cataracts. The P31150 protein (Rab GDP dissociation inhibitor-α) expression quantity was lower in all cataract types than in transparent lenses, but no variations were found between the different cataract types. The Q14240 protein (Eukaryotic Initiation Factor 4A2) was depleted in corticonuclear cataracts compared with transparent lenses and existed at about 1.68-fold reduced levels in corticonuclear than in cortical cataracts. The Q12934 protein (filensin) existed in decreased amounts in corticonuclear cataracts compared with transparent lenses, and corticonuclear cataracts also had a declined expression of this protein at about 1.51-fold lower than that in cortical cataracts. P04792 (Heat shock protein-β1) existed in lesser quantities in cortical, nuclear, and corticonuclear cataracts than in transparent lenses, but it did not differ between varying cataract types. 

### 3.5. Protein Differences According to Visual Acuity and Dysfunctional Lens Index

Supplementary Table S4 lists 78 proteins according to their correlation with visual acuity and the dysfunctional index.

Expectedly, protein correlations with visual acuity and dysfunctional lens index were either opposite or showed no statistically significant correlation with one parameter while correlating with the other. Ten proteins showing a positive correlation with the visual acuity were not negatively correlated with the dysfunctional lens index, and eleven proteins having a negative correlation with visual acuity were not positively correlated with the dysfunctional lens index. The discussion section only considers proteins exerting differences with *p*-values < 0.001 regarding visual acuity along with *p*-values < 0.05 regarding dysfunctional lens index. Nineteen proteins correlated significantly positively with visual acuity, where P80723, P07316, P22914, P04083, and P07320 constitute the top five that were always negatively correlated with the dysfunctional lens index. The number of proteins showing a negative correlation with visual acuity was higher than that of those with a positive correlation. Thirty-one proteins were correlated negatively with visual acuity with statistical significance, including 11 with *p*-values ≤ 0.005.

## 4. Discussion

Before discussing the findings particular to each protein, which show significant differences according to age, sex, lens/cataract features, visual acuity, and dysfunctional lens index, it is necessary to underline that this study can be considered only a proof-of-principle pilot study due to its small sample size. The age parameter showed the best range among these factors. At the same time, the sparsity of male patients (approximately 3.7 times lower than that of females) and the limited sample size (*n* = 4) of analyzed clear lenses constituted the most restricted samples. Therefore, special care was taken while commenting on the obtained results. Statistical significance with *p*-values lower than 0.05 was discussed regarding differences in patients’ sex, considering whether these proteins logically connect with cataractogenesis or exhibit additional connections with other parameters. For all the remaining correlations, only *p*-values less than 0.001 were considered in the discussion to minimize the confounding effects of low sample size.

### 4.1. Protein Differences According to Age

As described in the results section, several proteins correlating with age with robust significance also showed strong correlations with other parameters analyzed in this study. All of these proteins will not be discussed here if they form complexes with each other for their functioning or have very close functions, but rather in other relevant sections conjointly below to provide a cohesive view. First, the proteins that were negatively correlated with age will be discussed. Vimentin (UniProt ID: P08670) is an intermediate filament protein prominently expressed in lens fiber cells to connect the cytoskeleton to cell membranes, and one missense mutation encoding this protein causes pulverulent cataracts [5]. Vimentin undergoes degradation with calcium overload, which is among the causes of cortical cataracts [6]. Further, vimentin is very vulnerable to aging-associated protein damage mediated by disulfide oxidation, which concurs with the current finding regarding its negative correlation with age [7]. Catenin β1 (CTNB1, UniProt ID: P35222) is a cardinal regulator of cell adhesion, the development of optic neuroepithelium, and its loss causes aberrant differentiation of retinal epithelial cells to a ciliary phenotype [8,9]. The decline in CTNB1 may also be associated with an improper differentiation of epithelial cells in the lens’ transition zone, which would subsequently cause a perturbed protein composition and lens opacification. Supporting this prediction, activating the CTNB1/Wnt pathway in cultured corneal epithelia enhances epithelial cell compaction and quality [10]. LSAMP (Limbic system-associated membrane protein, UniProt: Q13449), named after its function for axon guidance in the cerebral limbic system, also has crucial functions for cytoskeletal organization [11]. Cilia are present in cells lining the intracerebral ventricles, in lens fiber cells, control intraocular fluid pressure, and their impaired vesicle trafficking causes Lowe Syndrome, which is associated with congenital cataracts, glaucoma, brain and other organ pathologies [2,12,13]. ADP Ribosylation Factor 3 (ARF3, UniProt ID: P61204) controls the trafficking and recycling of vesicles, proteins, and Flemming bodies regulating the cytoskeletal organization and cell division [14,15,16]. Hence, its decline may cause an accumulation of dysfunctional proteins and hamper cellular renewal in the lens’ transition zone. Carbonic Anhydrase 14 protein (CA14, UniProt ID: Q9ULX7) is a zinc-containing carbonic anhydrase (CA) isoenzyme. CAs control the eye’s water content; their inhibitors are used in glaucoma. Thus, CA enzymes may be considered harmful regarding cataractogenesis, but several findings indicate the opposite. Early studies showed that transparent, but not cataractous, lenses have high CA activity, even higher than that of the blood. This finding was attributed to CA enzymes supporting the lens, as the lens tissue relies on its metabolism due to a lack of microvascular nourishment [17]. The decline of CA14 in the aging lens may also be linked to the putative role of zinc in cataractogenesis, as CA14 levels decline in rat retinas with zinc deficiency [18]; and zinc-deficient diet causes cataracts in fish. This study also revealed two proteins that were positively correlated with age: the dynein axonemal heavy chain 9 (DNAH9, UniProt ID: Q9NYC9) and the forkhead-associated domain-containing protein 1 (FHAD1, UniProt ID: B1AJZ9). DNAH9 is a component of dyneins that construct the essential force for macromolecule and organelle transport toward the microtubules’ minus ends in all cell types, which project to the anterior pole in the lens [19]. Mutations in the *DYNC1H1* gene, encoding a protein with functions and a structure very similar to those of DNAH9, cause cataracts [20,21]. The accumulation of DNAH9 with aging may cause an aberrant increase in migration toward the lens’ anterior pole by fiber cells, which are regulated by dyneins, subsequently deteriorating the lens structure and increasing opacification. FHAD-containing transcription factors control embryonic patterning, mitosis, and DNA repair, and a lack of FHAD1 impairs sperm motility conceivably due to its role in the proper structuring of cilia [22,23,24]. The accumulation of FHAD1 with age may perturb ciliary structure and lens planar cell polarity, and may also be a response to aging-associated DNA injury.

### 4.2. Protein Differences According to Sex

Despite some controversies, global epidemiological studies mostly show higher cataract prevalence among women, and estrogen treatments for various indications enhance cataractogenesis [25,26]. There might be factors additional to estrogen that increase women’s vulnerability to cataractogenesis. The 4-hydroxyphenylpyruvate dioxygenase (4-HPD, UniProt ID: P32754), which was lower in women’s lenses, is a glyoxalase that catalyzes 4-hydroxyphenylpyruvate conversion to homogentisic acid. Glyoxalases detoxify dicarbonyls that damage cellular proteins, and despite the supraphysiological homogentisic acid causing a disease (alkaptonuria), it is also a potent antioxidant [27,28]. Therefore, lower 4-HPD may augment the vulnerability of lenses to protein crosslinking and deprive them of an endogenous antioxidant. Castration lowers, and testosterone substitution elevates circulating 4-HPD in men [29]. This testosterone feature may cause relatively higher levels of an enzyme with putative protective functions in the lenses of males. Lower caveolin 1 (CAV1, UniProt ID: Q03135) levels in women’s lenses may also be associated with the negative correlation between age and CAVIN (Caveolae-associated protein 1) found in this study. CAV1 needs the accompaniment of CAVIN to form caveolae, cell membrane endocytosis organelles, and it depletes during the degradation of lens epithelial cell membranes under oxidative and glucose stress [30,31,32]. On a rare occasion of CAV1-encoding gene mutation affecting siblings of one boy and one girl, only the boy developed cataracts, suggesting the likelihood that CAV1 has a superior role in protecting males against cataracts, in parallel to current results [33]. CAV1 and CAVIN companions may accelerate endosomal trafficking and the renewal of damaged intracellular content in the lens, and both may deplete due to relatively lower metabolic defense in women’s lenses and aging, respectively. Heat shock protein HSP 90-β (HS90B, UniProt ID: P08238), which was lower in women’s lenses, is a member of the chaperone family that repairs structurally damaged proteins or degrades them if they are irreversibly damaged, binds to the receptor of the mainly female and cytoprotective hormone, progesterone, and mediates caveolin transport into lysosomes [34,35]. These features hint at ternary interactions between HS90B, caveolin, and cytoprotective hormones, where reductions in the components of these interactions may cause the accumulation of damaged proteins and cataractogenesis. The proteins that exist more in women’s lenses will be discussed now. The Rab GDP dissociation inhibitor-β (RAB8B, UniProt ID: P50395) is encoded by the RAB8B gene, whose gain-of-function variant enhancing RAB8B protein levels increases schizophrenia risk [36]. Diabetics and celiac disease patients develop antibodies against RAB8B, and diabetic patient serum stimulates mesenchymal cell RAB8B expression, indicating that high levels of RAB8B potentially trigger vicious autoinflammatory and cytotoxic cycles [37,38]. The brain isoform of V-type proton ATPase subunit B (ATP6V1B1, UniProt ID: P21281) translocates protons and strongly acidifies the intracellular and extracellular milieu [39,40]. One of the oxidative modifications of lens crystallins is acidification, and β-crystallin deamidation during aging, in turn, acidifies proteins [4]. Lactic acid is continuously produced within the lens due to the primary utilization of anaerobic glycolysis for producing energy [41]. All these factors indicate the likelihood that high levels of a robustly acidifying protein may harm the homeostasis of the lens, whose structural components are already vulnerable to acidity.

Having provided plausible explanations for sex-dependent differences in specific lens proteins, it is necessary to acknowledge that there is a considerable imbalance in the sex distribution of the study cohort, where female patients constituted approximately 3.7 times the percentage of males. This fact precludes making very firm conclusions. Still, the observed differences will be of value if confirmed in subsequent studies, as these proteins cluster within closely related functions.

### 4.3. Protein Differences According to Lens and Cataract Features

This research revealed that cataractous lenses have lower levels of many proteins than transparent lenses, among six whose expressions differed with *p*-values below 0.001 will be discussed. Yet, only corticonuclear-type cataracts significantly expressed lesser proteins likely with putative protective functions, which always occurred when compared with cortical cataracts. The lactase-like protein (LCTL, UniProt ID: Q6UWM7) quantity was lower in all cataracts except for the cortical type. LCTL maintains eye lens suture formation, and it was shown to hydrolyze amino acid glycosidic bonds in mice expressing a human analog LCTL protein [42]. Low LCTL levels in the lens may cause suboptimal removal of protein glucose adducts and accumulation of advanced glycation endproducts, contributing to cataractogenesis, which is coherent with another finding of this study, showing age-related declines in LCTL. Rab GDP dissociation inhibitor-α (GDI, UniProt ID: P31150) regulates vesicular membrane trafficking, and endothelial cells boost GDI expression after exposure to powerful oxidants [43]. Mice deficient in UCH L1 enzyme, involved in damaged protein removal, develop neurodegeneration accompanied by oxidatively modified GDI accumulation [44]. Many antioxidant defense proteins are themselves vulnerable to oxidation, which also explains GDI depletion in cataractous lenses, as persistent oxidative injury is among the foremost components of cataractogenesis. Phakinin (UniProt ID: Q13515) and filensin (UniProt ID: Q12934) were lower in cataracts than in transparent lenses and negatively correlated with age, in complete parallelity with prior studies. Phakinin and filensin bind together to form beaded lens filaments, and rise throughout life together with crystallins for packaging fiber cells by ascending toward the remodeling zone, where lens cells can synthesize proteins and renew [45]. In animals deficient in beaded filaments, the lens loses its quality and forms hazes. Phakinin levels are reduced in nuclear cataracts, and its encoding gene (BFSP2) mutations cause juvenile-onset cataracts [46,47]. Mutations encoding filensin (BFSP1) also induce congenital cataracts, and in rat models with excess selenium-induced oxidative cataractogenesis, filensin expression reduces in the lens, which is preventable with antioxidants [48,49]. Loss-of-function gene mutations encoding heat shock protein-β1 (HSPB1, UniProt ID: P04792) cause human lamellar cataracts, and its levels increase in lens tissue cultures upon exposure to high-dose glucocorticoids that bind to nucleophilic amino acids and destabilize proteins, which is responsible for posterior subcapsular cataract development after steroid treatment [50]. The increase in HSPB1 with steroid exposure may be a fast protective response. Still, it may not hinder the clearance of proteins damaged due to life-long endogenous steroid exposure, which subsequently causes its decline in cataracts. Eukaryotic Initiation Factor 4A2 (EIF4A2, UniProt ID: Q14240) was another protein lower in cataractous lenses and correlated negatively with age together with a structurally and functionally very similar protein, EIF4A1. EIF4A2-encoding gene mutations cause epilepsy and cerebral anomalies in humans, and transfecting Drosophila with these mutations perturbs decapentaplegic signaling, which regulates eye development [51]. As cells in the lens’ transitional zone retain some embryonic features, they may still depend on EIF4A2 for renewal and lens repair [3]. High-glutamate-induced cerebral damage in rats, which involves oxidative and nitrosative injury, enhances the EIF4A2 protein [52]. This feature may be due to the conceivably antioxidative functions of EIF4A2, a likelihood strengthened by structurally very similar EIF4A1 protein conjugates with toxic lipid peroxidation products [53]. As proposed for HSPB1 above, EIF4A1 and EIF4A2 may not compensate for the persistence of hazardous intermediates, leading to their depletion with the completion of cataractogenesis. The plausible reasons why the reductions in the likely lens-preserving proteins happened consistently in corticonuclear cataracts and why the cortical cataracts were less affected deserve discussion. As their name implies, corticonuclear cataracts involve the damage of two different lens compartments, supposedly due to more severe molecular and cellular damage, yet to be defined. The lens-covering epithelia are primarily responsible for producing the antioxidant glutathione that prevents protein damage, which may be associated with lower declines in protective proteins in cortical cataracts [3,54].

### 4.4. Protein Differences According to Visual Acuity and Dysfunctional Lens Index

#### 4.4.1. Proteins Positively Correlating with Visual Acuity

Brain Acid Soluble Protein-1 (BASP1, UniProt ID: P80723) had the highest positive and negative correlations with visual acuity and the dysfunctional lens index, respectively. The BASP1 protein resides within the neuron axons to regulate the cytoskeleton, and its levels increase from the lens’ differentiation to the remodeling zone together with phakinin and filensin [45]. Likely, BASP1 cooperates with filament proteins to maintain lens qualities, providing adequate visual acuity. Annexin A1 (ANXA1, UniProt ID: P04083), a cell membrane phospholipid-binding antiinflammatory protein, was also correlated notably positively with visual acuity and negatively with the dysfunctional lens index. A connexin gene-deficient mouse strain develops cataracts, which become less susceptible to cataractogenesis if they carry a specific ANXA1 variant [55]. Cataract and glaucoma pathogeneses may partially overlap, as atherosclerosis and lowered eye blood supply increase intraocular pressure, which may deteriorate lens microcirculation, antioxidant transport, and the removal of toxic cellular waste [56]. Proteomic comparisons of cataract patients with or without acute primary angle-closure glaucoma (APAG) revealed that patients having both pathologies had the highest ANXA1 levels [57]. These findings provoke the idea that ANXA1 possesses protective functions in the lens. Dynein Heavy Chain Domain-Containing Protein-1 (DNHD1, UniProt ID: Q96M86) may relate to dynein functions in the trafficking of endolysosomes and autophagosomes, degrading damaged proteins [58,59]. Hence, DNHD1 may help prevent toxic protein accumulation to maintain lens quality, which could be a reason for the positive association of visual acuity with DNHD1. Similar to the antecedent studies for phakinin and filensin, the associations of lens crystallins with visual acuity were consistent with former results. Besides reducing light scattering, lens crystallins act as chaperones, as heat shock proteins do. Mutations in the gene encoding Gamma-crystallin D protein (CRGD, UniProt ID: P07320) cause human cataracts and d-asparagine residues in the CRGD protein increase in rabbit lenses after infrared light exposure, suggesting potential functions of CRGD against cataracts [60,61]. Gamma-crystallin B (CRGB, UniProt ID: P07316) is a member of evolutionary conserved γ-crystallins, and mutations or variations in CRGB-encoding gene cause autosomal dominant congenital cataracts and sporadic pediatric cataracts, respectively [62,63]. Two proteins, β-crystallin B (CRYBB1, UniProt ID: P53674) and γ-crystallin S (CRYGS, Uniprot ID: P22914), that were positively correlated with age in the current investigation were also correlated positively with visual acuity. These findings may seem antithetical, but could be explained by the foremost roles of CRYBB1 and CRYGS in protecting the lens against noxious stimuli (i.e., oxidative stress), causing damage and accumulation due to life-long exposure to harmful metabolites. Supporting this presumption, CRYBB1 increases with age, diabetes, and after cataract surgery, and exists at higher levels in the posterior parts of the lens, where the absorption of ultraviolet B and C waves is higher [64,65,66]. Vice versa, the expression of the CRYBB1-encoding gene is lower in vervet monkeys developing congenital cataracts [67]. Gene mutations of CRYGS, an essential protein for lens transparency, also cause congenital lamellar cataracts in humans [68].

#### 4.4.2. Proteins Negatively Correlating with Visual Acuity

Ribose–phosphate pyrophosphokinase-1 protein (PRPS1, UniProt ID: P60891) had the highest negative correlation with visual acuity, which was positively correlated with the dysfunctional lens index, which is essential for purine synthesis and increases in its transcription or enzymatic activity elevate urea levels [69,70]. Cataracts are associated with higher uric acid levels during aging, and in preclinical models, excess uric acid causes cellular senescence and opacification in the lens [71]. Therefore, high PRPS1 levels may cause cataractogenesis through uric acid overproduction, which may link to its negative correlation with visual acuity. SEC14-like protein 2 (SEC14L2, Uniprot ID: O76054) binds phospholipids and α-tocopherol, and belongs to the protein family of the supernatant protein factor (SPF), which converts squalene to cholesterol that exists at highest levels in the lens among all human tissues and is generally appraised as protective [72,73]. However, cholesterol also causes a robust stiffness of cell membranes and inhibits SERCA (endoplasmic reticulum Na/Ca exchanger) when lens lipids’ hydrocarbon chains become disordered [73]. Hence, excess cholesterol may be detrimental to the lens instead of beneficial. People with a SEC14L2-encoding gene variant have a higher glaucoma risk [74]. If this variant causes a SEC14L2 gain-of-function, it can boost cholesterol synthesis, accelerate atherosclerosis, and raise intraocular pressure, leading to glaucoma and impaired lens microcirculation. T-complex protein-1 subunit-δ (TCPD, UniProt ID: P50991), which was correlated negatively with the visual acuity, is a chaperone protein, a component of telomerase TriC complex, and binds to the native and denatured forms of actin [75,76,77]. At first glance, this finding seems paradoxical, since chaperones repair or remove damaged proteins, and the telomerase enzyme mediates cellular renewal. Yet, if TCPD is involved in compensating protein damage throughout life, ongoing oxidation and amidation events may induce a harmful accumulation of itself and denatured actin. Further, telomerase activity gradually declines with age, a physiological mechanism against pathological cell overgrowth. Hence, the overactivity of telomerase may cause aberrant proliferation of renewable cells in the remodeling zone under the lens capsule, which may lead to the lens’s thickening and dysfunction.

1. A Conceptual Framework for Cataractogenesis

**Figure 1:**
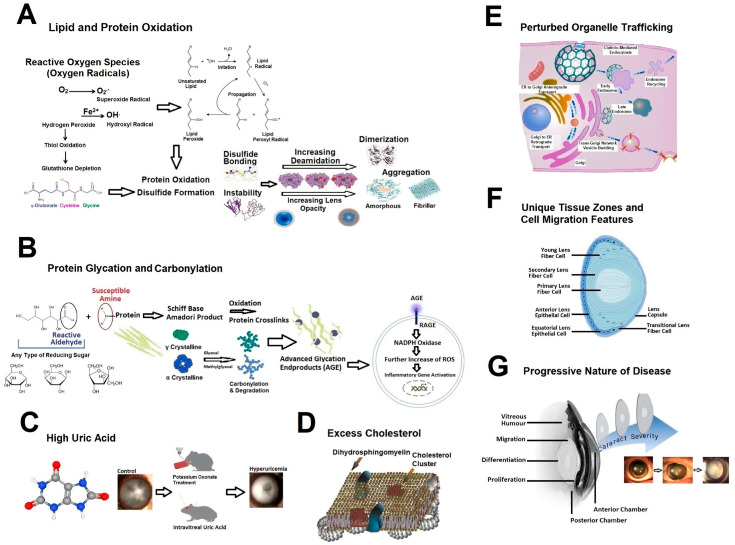
A conceptual framework for cataract pathogenesis. (**A**) ROS oxidize lipids and proteins directly and indirectly through the lipid peroxides. Hydrogen peroxide depletes glutathione. All these events cause protein disulfides, dimerization, and aggregation accelerated by deamination (adapted from Norton-Baker et al. [78]). (**B**) Lens proteins are harmfully modified by glycation with reducing sugars and by carbonylation with carbonyl products. The sugars’ aldehyde and proteins’ amine groups form Schiff bases and Amadori products, whose oxidation produces advanced glycation end products (AGEs), activate their membrane receptors (RAGEs). This activation triggers NADPH oxidase to produce further ROS. (**C**) High uric acid induces cataracts, as revealed by feeding rats with a uricase inhibitor or intravitreal injection of uric acid (adapted from Lin et al. [71]). (**D**) Cholesterol is generally considered protective, but its excess may be detrimental through atherosclerosis and hampering lens microcirculation (adapted from Borchman and Yappert [73]). (**E**) Most of the proteins that declined with age are involved in structuring the cell cytoskeleton, indicating a possible role of aberrant organelle trafficking in cataractogenesis (adapted from https://www.reactome.org/content/detail/R-HSA-199991, accessed on 15 April 2025). (**F**) Unique tissue zones and cell migration events, such as those in the remodeling zone, could make the lens vulnerable to disturbed proliferative signals by the dysregulated cytoskeleton and telomerase complex. (**G**) Cataract is a dynamic pathology where multiple factors mutually interact and propagate detrimental effects of each other.

Based on current and previous results, Figure 1 and its subfigures depict a conceptual framework for cataract pathogenesis.

Reactive oxygen species (ROS) constitute a major risk, which are overproduced due to several conditions, such as smoking, UV light, and aging (Figure 1A). ROS deplete glutathione and oxidize lipids and proteins, causing protein aggregation, which is accelerated by deamidation. Especially with diabetes, glycation and carbonylation perturb the structure of lens proteins, and signals triggered by advanced glycation endproducts (AGE) and their receptors (RAGE) further injure the composition of lenses (Figure 1B). High uric acid may be another risk factor through enhancing cell senescence (Figure 1C). After a certain threshold, cholesterol may act detrimentally, rather than beneficially (Figure 1D). Perturbed organelle trafficking emerges as a conceivable component of cataractogenesis, as most aging-associated lens proteins involve organelle transport (Figure 1E). Unique lens zones comprising proliferative and migratory cells may also destine the lens to the harmful effects of specific protein changes (Figure 1F). Lastly, many etiopathological factors converge in cataract pathogenesis, which could mutually accelerate cataract growth (Figure 1G).

2. Limitations and Strengths

The main limitation of this study is the small number of patients analyzed, which precludes the analysis of confounding factors, such as smoking, UV light exposure, and metabolic diseases like diabetes. The number of transparent lenses used as controls is low. Still, transparent lenses not influenced by any pathology may have a much more homogenous protein profile, enabling them to be used for comparisons. Further, as mentioned in the discussion, regarding four of the five statistical analyses employed, the outcomes with the highest significance (*p* < 0.001) were interpreted to provide the most relevant and scientifically solid inferences that could be obtained from a low sample size. Despite females being represented approximately 3.7 times more than males in this study, sex-associated changes with *p*-values below 0.05 were discussed. The reason for this is that there are almost proteomics studies about the role of sex in cataractogenesis, while several large-scale epidemiological studies showed that females had a higher risk of developing cataracts. Hence, the aim was to engage the attention of scientists working in this field on these proteins, considering that sex-associated differences also followed a relatively consistent functional pattern. Nonetheless, the results regarding sex differences still need to be evaluated with caution, as low sample numbers may have caused shifts in *p*-values in either direction. This study’s strength is that all patients were diagnosed and followed up by the same ophthalmologist in the same specialized clinic. Also, the concurrency of current results with previous findings regarding filensin, phakinin, and crystallins supports the reliability of the obtained data.

## 5. Conclusions

This study found hitherto unreported proteins associated with cataractogenesis that include chaperons, proteins regulating the trafficking of organelles removing damaged proteins, glyoxalases detoxifying dicarbonyls, and an enzyme producing uric acid, recently shown as a contributor to cataract formation. Considering the significant overlap of several other findings of the current investigation with previous reports, these novel observations deserve further research.

## Figures and Tables

**Table 1:** Demographic and clinical features.

**Number of Patients**	**33**
**Age**	
Mean ± STD Median (min–max)	62.08 ± 9.32 63.5 (45–85)
	
**Sex**	***n* (%)**
Female	26 (78.79)
Male	7 (21.21)
**Lens or Cataract Type**	
Transparent Lens	4 (12.12)
Cortical Cataract	5 (15.15)
Nuclear Cataract	9 (27.27)
Corticonuclear Cataract	10 (30.30)
	
Other	5 (15.15)
	
	**Mean ± STD**
**Visual Acuity (V-CC LogMar)**	0.26 ± 0.25
**Dysfunctional Lens Index (DLI)**	5.38 ± 2.76

**Table 2:** Age, dysfunctional lens index (DLI), and visual acuity (V-CC LogMar).

Lens Type	Age (Mean ± STD) (Min–Max)	DLI (Mean ± STD) (Min–Max)	V-CC (Mean ± STD) (Min–Max)
**Cortical** **Cataract**	57 ± 2.6 (51–66)	7.09 ± 1.43 (3.61–10.0)	0.5 ± 0.01 (0.00–0.1)
**Nuclear** **Cataract**	66.78 ± 2.54 (54–75)	6.09 ± 0.82 (2.36–9.32)	0.21 ± 0.04 (0.05–0.40)
**Corticonuclear Cataract**	73.90 ± 1.90 (66–85)	3.57 ± 0.85 (1.31–8.97)	0.41 ± 0.07 (0.15–0.80)
**Mixed Cataract**	63.22 ± 3.81 (45–78)	4.07 ± 0.76 (1.54–7.11)	0.43 ± 0.10 (0.00–1.00)
**Clear Lens**	49.50 ± 2.02 (46–55)	7.51 ± 0.76 (5.67–9.08)	0.012 ± 0.001 (0.00–0.05)

**STD:** Standard Deviation.

## Data Availability

Data are available upon reasonable request from the corresponding author.

## Supplementary Materials

The following supporting information can be downloaded at: https://www.mdpi.com/article/10.3390/jcm14134737/s1, Table S1: Proteins Correlating with Age; Table S2: Proteins Exerting Sex-Dependent Differences in Expression; Table S3: Protein Levels Classified According to Cataract or Lens Features; Table S4: Proteins Classified According to Visual Acuity and Dysfunctional Lens Index.
