# Supplementary material for: Lens Proteomics Provide Novel Clues for Cataractogenesis: Original Investigation and a Broad Literature Survey
Source: J Clin Med. 2025 Jul 4;14(13):4737. doi: 10.3390/jcm14134737 (PMC12251298; doi:10.3390/jcm14134737)
Supplement: Supplementary file 1 [file jcm-14-04737-s001.zip › jcm-3635661-supplementary.pdf]

**Supplementary Table S1.** Proteins Correlating with Age

| Protein | r | p | Protein | r | p | Protein | r | p | Protein | r | p |
|---|---|---|---|---|---|---|---|---|---|---|---|
| P13489 | **-0.492** | **0.004** | P07196 | -0.307 | 0.082 | O15020 | **0.416** | **0.016** | P60842 | **-0.583** | **<0.001** |
| Q9H4G0 | **-0.439** | **0.011** | Q9H1H9 | -0.164 | 0.362 | P18085 | **-0.559** | **0.001** | P50395 | **-0.457** | **0.007** |
| Q6NZI2 | **-0.603** | **<0.001** | Q9Y6H8 | **-0.355** | **0.043** | P11844 | **0.344** | **0.050** | P30041 | **-0.506** | **0.003** |
| Q6ZMZ3 | 0.330 | 0.061 | P53672 | **-0.410** | **0.018** | P07205 | **0.527** | **0.002** | P07316 | **0.408** | **0.018** |
| Q9NYC9 | **0.765** | **<0.001** | P80723 | **0.546** | **0.001** | P50991 | **-0.569** | **0.001** | P68032 | **0.351** | **0.045** |
| P21281 | **-0.500** | **0.003** | P23528 | 0.215 | 0.229 | O75828 | **0.435** | **0.011** | P08238 | 0.129 | 0.473 |
| B1AJZ9 | **0.580** | **<0.001** | P35222 | **-0.651** | **<0.001** | P26038 | **-0.496** | **0.003** | P11216 | 0.309 | 0.080 |
| P14550 | **0.487** | **0.004** | P55064 | -0.200 | 0.265 | Q99497 | 0.022 | 0.903 | P07315 | **0.425** | **0.014** |
| Q13449 | **-0.702** | **<0.001** | P04083 | **0.529** | **0.002** | P30086 | **0.466** | **0.006** | P08670 | **-0.647** | **<0.001** |
| Q9BYB0 | **-0.434** | **0.012** | Q8N9V7 | 0.283 | 0.110 | P32754 | 0.003 | 0.986 | P07320 | **0.476** | **0.005** |
| P00387 | **-0.528** | **0.002** | Q13315 | **-0.497** | **0.003** | P11171 | **-0.442** | **0.010** | P04406 | **-0.356** | **0.042** |
| O76054 | **-0.560** | **0.001** | Q06830 | -0.282 | 0.112 | P38606 | **-0.441** | **0.010** | P22914 | **0.574** | **<0.001** |
| P34913 | -0.286 | 0.106 | Q03135 | **-0.395** | **0.023** | P11413 | **-0.413** | **0.017** | P53674 | **0.697** | **<0.001** |
| P22748 | **-0.397** | **0.022** | P60891 | **-0.557** | **0.001** | Q6UWM7 | **-0.689** | **<0.001** | Q12934 | **-0.723** | **<0.001** |
| P13645 | -0.250 | 0.160 | Q9Y281 | -0.212 | 0.237 | P07437 | **-0.445** | **0.009** | Q13515 | **-0.774** | **<0.001** |
| P63000 | -0.262 | 0.140 | P06744 | -0.214 | 0.232 | P22061 | **-0.356** | **0.042** | | | |
| Q99832 | -0.306 | 0.083 | P36405 | -0.127 | 0.482 | P18669 | -0.200 | 0.266 | | | |
| P27361 | -0.141 | 0.433 | P00918 | 0.189 | 0.291 | P04792 | **-0.504** | **0.003** | | | |
| P60981 | **-0.471** | **0.006** | P61204 | **-0.614** | **<0.001** | Q9NY65 | **0.516** | **0.002** | | | |
| O15498 | -0.241 | 0.178 | Q14690 | **-0.412** | **0.017** | P31150 | **-0.519** | **0.002** | | | |
| Q9ULX7 | **-0.635** | **<0.001** | P48163 | **0.380** | **0.029** | Q14240 | **-0.738** | **<0.001** | | | |
| Q96M86 | **0.492** | **0.004** | P04264 | -0.114 | 0.526 | P04075 | **0.460** | **0.007** | | | |

* r= Correlation Coefficient. p= Significance.

**Supplementary Table S2.** Proteins Exerting Sex-Dependent Differences in Expression

| Protein | Sex | | P value | Protein | Sex | | P Value |
|---|---|---|---|---|---|---|---|
| | Female | Male | | | Female | Male | |
| | Mean ± STD | Mean ± STD | | | Mean ± STD | Mean ± STD | |
| P13489 | 180.31±131.79 | 304.95±239.78 | 0.228 | Q14690 | 5,307.59±2,417.32 | 4,612.1±1,758.09 | 0.484 |
| Q9H4G0 | 500.68±238.17 | 612.97±201.78 | 0.264 | P48163 | 11,3491.63±44,057.89 | 94,334.12±62,419.47 | 0.469 |
| Q6NZI2 | 570.59±380.72 | 483.94±261.6 | 0.577 | P04264 | 1,381.14±1,376.33 | 1,158.81±474.41 | 0.680 |
| Q6ZMZ3 | 3,430.99±1361.03 | 3,352.93±1,155.17 | 0.891 | O15020 | 727.24±719.59 | 1,103±1,251.33 | 0.471 |
| Q9NYC9 | 35,065.7±17,577.11 | 37,282.21±19,506.6 | 0.774 | P18085 | 1,469.23±732.89 | 1,647.31±824.72 | 0.582 |
| **P21281** | **2,296.69±1,395.39** | **840.65±812.69** | **0.013** | P11844 | 38,884±22,386.34 | 27,938.57±24,382.59 | 0.268 |
| B1AJZ9 | 1,973.54±1,122.13 | 2,882.91±1,628.66 | 0.094 | P07205 | 3,456.69±1,928.52 | 3,411.2±1,714.58 | 0.955 |
| P14550 | 2,532.77±1,200.58 | 2,802.69±487.9 | 0.568 | P50991 | 4,836.15±1,665.6 | 4,039.9±1,359.03 | 0.255 |
| Q13449 | 1,883.3±1,106.17 | 1,830.55±1,141.65 | 0.912 | O75828 | 69,510.33±25,314.8 | 74,504.21±32,575.04 | 0.666 |
| Q9BYB0 | 9,438.84±2,910.11 | 7,487.46±3,258.89 | 0.134 | P26038 | 3,204.16±1,428.67 | 3,561.45±1,407.29 | 0.560 |
| P00387 | 579.12±384.38 | 612.49±468.68 | 0.847 | Q99497 | 62,449.45±26,644.46 | 84,110.42±32,887.23 | 0.079 |
| O76054 | 1,101.77±496.23 | 1,121.39±612.51 | 0.930 | P30086 | 10,228.7±3,419.11 | 10,466.49±2,536.45 | 0.865 |
| P34913 | 929.79±511.92 | 1,118.21±781.97 | 0.564 | **P32754** | **7,449.16±2,852.01** | **12,056.84±5,052.94** | **0.003** |
| P22748 | 1,331.83±650.94 | 1,595.17±657.33 | 0.350 | P11171 | 11,270.13±2,774.92 | 12,030.21±3,495.12 | 0.547 |
| P13645 | 1,351.96±649.95 | 1,575.21±515.56 | 0.409 | P38606 | 5,669.84±1,836.87 | 6,527.07±2,129.8 | 0.297 |
| P63000 | 5,076.7±1,155.94 | 6,775.89±2,500.63 | 0.126 | P11413 | 8,448.36±2,213.96 | 8,708.41±3,268.46 | 0.805 |
| Q99832 | 1,773.12±962.8 | 1,793.3±974.41 | 0.961 | Q6UWM7 | 16,643.74±6,969.37 | 16,398.97±5,791.84 | 0.933 |
| P27361 | 890.23±427.3 | 1,127.85±386.06 | 0.193 | P07437 | 1,277.85±708 | 1,378.72±689.48 | 0.739 |
| P60981 | 2,476.79±766.68 | 2,582.65±754.07 | 0.747 | P22061 | 13,799.18±4,982.7 | 18,076.95±8,805.96 | 0.257 |
| O15498 | 2,654.23±641.56 | 2,503.5±508.96 | 0.571 | P18669 | 3,604.65±1,495.96 | 6,652.73±4,482.07 | 0.124 |
| Q9ULX7 | 4,704.04±2,299.21 | 5,427.95±2,600.55 | 0.477 | P04792 | 56,449.01±27,903.83 | 67,055.16±29,789.54 | 0.385 |
| Q96M86 | 20,918.48±12,370.97 | 22,808.28±14,076.76 | 0.729 | Q9NY65 | 4,136.07±1,592.46 | 4,809.44±2,062.92 | 0.358 |
| P07196 | 5,440.77±1,421.12 | 6,349.44±2,675.53 | 0.415 | P31150 | 23,458.55±12,906.12 | 19,749±5,266.93 | 0.467 |
| Q9H1H9 | 61,879.04±40,047.49 | 31,728.6±33,939.99 | 0.079 | Q14240 | 1,718.55±499.89 | 1,952.25±790.38 | 0.341 |
| Q9Y6H8 | 7,001.09±2,339.71 | 8,602.48±2,740.58 | 0.131 | P04075 | 107,513.8±40,150.76 | 10,5490.83±28,819.59 | 0.902 |
| P53672 | 15,838.84±7,771.36 | 21,096.35±10,771.39 | 0.153 | P60842 | 11,795.89±4,049.85 | 9,096.09±2,022.87 | 0.100 |
| P80723 | 14,522.18±9,509.7 | 18,078.19±12,982.44 | 0.423 | **P50395** | **39,429.85±17,979.19** | **22,409.6±13,620.72** | **0.027** |
| P23528 | 1,542.72±10,78.9 | 1,275.56±1,345.11 | 0.584 | P30041 | 67,198.25±30,743.15 | 67,625.95±16,065.03 | 0.972 |
| P35222 | 2,291.3±776.8 | 2,837.16±1,047.41 | 0.135 | P07316 | 50,079.21±23,901.13 | 61,204.65±20,966.03 | 0.272 |
| P55064 | 8,457.61±5,569.69 | 11,461.66±8,411.58 | 0.266 | P68032 | 7,562.05±4,350.23 | 11,315.7±11,436.86 | 0.425 |
| P04083 | 7,810.8±7,039.98 | 7,918.17±7,974.77 | 0.972 | **P08238** | **42,107±20,247.23** | **61,542.9±19,823.95** | **0.031** |
| Q8N9V7 | 42,907.94±28,225.17 | 49,305.53±16,611.37 | 0.573 | P11216 | 11,6754.67±47,492.92 | 131,385.21±42,095.14 | 0.465 |
| Q13315 | 2,347.42±1,003.38 | 2,889.71±1,514.1 | 0.264 | P07315 | 557,028.77±213,676.93 | 520,457.44±215,496.75 | 0.691 |
| Q06830 | 918.8±372.69 | 1,395.58±796.98 | 0.170 | P08670 | 62,755.21±35,852.98 | 74,109.59±43,348.11 | 0.481 |
| **Q03135** | **6,023.25±2,555.31** | **8,692.79±4,030.3** | **0.038** | P07320 | 928,624.01±386,259.53 | 901,745.31±306,421.51 | 0.866 |
| P60891 | 2,048.46±824.71 | 1,932.77±864.39 | 0.746 | P04406 | 510,456.01±246,235.13 | 536,990.82±227,885.19 | 0.799 |
| Q9Y281 | 9,589.73±3,652.62 | 8,294.18±2,273.6 | 0.382 | P22914 | 2,098,419.19±94,2021.39 | 2,382,734.99±957,985.35 | 0.485 |
| P06744 | 2,175.5±884.78 | 1,759.38±793.47 | 0.269 | P53674 | 2,860,235.91±807,466.78 | 3,297,712.96±850,685.3 | 0.217 |
| P36405 | 1,944.72±557.86 | 2,354.92±815.17 | 0.128 | Q12934 | 1,129,428.23±30,6567.7 | 1,173,879.59±376,435.76 | 0.747 |
| P00918 | 6,641.03±2,626.71 | 5,557.08±892.75 | 0.295 | Q13515 | 1,500,338.27±45,7879.26 | 1,476,776.91±447,546.94 | 0.904 |
| P61204 | 3,094.12±1,477.96 | 3,123.19±1,446.69 | 0.963 | | | | |

**STD:** Standard Deviation. The proteins demonstrating statistically significant changes were written in bold letters.

**Supplementary Table S3.** Protein Levels Classified According to Cataract or Lens Features

| Protein | Lens or Cataract Type | | | | | | |
|---|---|---|---|---|---|---|---|
| | 0: Transparent Lens | 1: Cortical | 2: Nuclear | 3: Corticonuclear | 4: Mixed Type | | |
| | Mean±STD | Mean±STD | Mean±STD | Mean±STD | Mean±STD | P Value | Difference |
| P13489 | 321.28±224.46 | 255.1±176.56 | 195.51±165.1 | 133.03±99.5 | 234.46±198.96 | 0.347 | |
| Q9H4G0 | 677.74±266.64 | 676.07±272.05 | 461.2±163.22 | 401.74±197.3 | 609.8±238.34 | 0.081 | |
| **Q6NZI2** | **1,083.09±610.59** | **587.81±353.59** | **503.34±190.08** | **347.07±211.38** | **590.17±213.71** | **0.007** | 0-2, 0-3 |
| Q6ZMZ3 | 2,121.48±434.56 | 3,484.69±1,778.32 | 3,173.48±966.66 | 3,756.69±1,221.06 | 4,127.74±1,523.97 | 0.164 | |
| Q9NYC9 | **18,760.45±5,374.85** | **27,564.51±10,868** | **32,689.1±10,993.93** | **49,312.93±21,916.39** | **34,497.65±13,831.35** | **0.017** | 0-3 |
| P21281 | **3,970.65±1,355.06** | **1,869.86±1,654.99** | **2,231.79±1,176.63** | **1,322.24±1,086.53** | **1,411.64±967.8** | **0.015** | 0-3, 0-4 |
| B1AJZ9 | 1,200.72±284.28 | 1,863.83±783.4 | 1,981.82±986.49 | 2,653.62±1,863.08 | 2,599.56±778.9 | 0.318 | |
| **P14550** | **1,364.97±566.04** | **2,215.83±473.79** | **2,431.64±1,002.12** | **3,161.9±1,135.64** | **3,085.64±1,111.03** | **0.034** | 0-3 |
| **Q13449** | **3,154.85±1,676.84** | **2,305.08±765.97** | **1,974.71±819.88** | **991.41±376.78** | **1,989.68±1,177.81** | **0.005** | 0-3 |
| Q9BYB0 | 12,032.27±1,021.61 | 9,661.63±654.75 | 9,131.18±2,594.47 | 7,447.55±3,070.63 | 8,945.76±4,734.7 | 0.139 | |
| **P00387** | **1,126.96±483.26** | **770.69±494.61** | **499.14±214.25** | **338.95±145.12** | **620.34±434.89** | **0.005** | 0-2, 0-3 |
| **O76054** | **1,409.16±227.63** | **1,473.21±332.92** | **1,325.91±442.2** | **639.62±389.17** | **1,032.72±548.65** | **0.002** | 0-3, 1-3, 2-3 |
| P34913 | 1,220.36±543.6 | 1,225.94±682.26 | 1,004.57±582.77 | 727.07±520 | 935.82±577.14 | 0.478 | |
| P22748 | 1,754.45±682.75 | 1,702.88±1018.66 | 1,531.62±606.86 | 1,023.01±370.52 | 1,249.39±545.49 | 0.179 | |
| P13645 | 1,600.46±485.56 | 1,636.46±588.46 | 1,298.34±472.42 | 1,265.88±887.78 | 1,449.91±428.29 | 0.783 | |
| P63000 | 5,741.08±1539.47 | 6,269.47±2,102.8 | 5,366.54±1293.1 | 4,361.85±992.65 | 6,639.26±2,069.99 | 0.067 | |
| Q99832 | 1,969.81±327.89 | 2,028.83±784.13 | 1,575.37±354.78 | 17,25.85±1,576 | 1,838.78±680.82 | 0.925 | |
| P27361 | 860.98±111.54 | 1,252.51±520.33 | 910.59±367.83 | 822.12±461.62 | 983.58±495.03 | 0.467 | |
| P60981 | 3,368.06±820.25 | 2,729.82±770 | 2,360.32±646.88 | 2,138.47±745.44 | 2,545.24±405.78 | 0.066 | |
| O15498 | 2,742.35±739.58 | 2,823.66±462.33 | 2,742.31±544.86 | 2,427.22±756.12 | 2,498.76±525.91 | 0.713 | |
| **Q9ULX7** | **7,737.63±3,615.13** | **6,417.2±2,646.35** | **4,643.11±1,395.16** | **3,110.76±1,004.46** | **4,873.7±1,367.85** | **0.002** | 0-3, 1-3 |
| Q96M86 | 10,673.51±871.4 | 13,440.76±5,870.59 | 21,070.05±9,029.51 | 27,576.44±16,419.12 | 25,649.14±11,900.34 | 0.082 | |
| P07196 | 5,426.86±2,254.25 | 6,283.81±1,777.29 | 5,485.46±1,289.06 | 4,844.94±1,329.44 | 6,992.24±2,396.97 | 0.210 | |
| Q9H1H9 | 86,280.9±58,397.39 | 59,790.32±38,356.31 | 55,747.13±42,332.61 | 59,464.65±31,136.14 | 18,101.84±21,273.57 | 0.141 | |
| **Q9Y6H8** | **10,013.93±1,337.31** | **8,427.17±2,858.81** | **7,141.39±1,887.24** | **5,558.85±1,404.64** | **8,038.63±3,268.32** | **0.014** | 0-3 |
| P53672 | 23,091.44±14,100.79 | 19,064.93±6,900.53 | 14,766.01±9,657.01 | 14,303.31±5,084.13 | 19,173.34±8,394.08 | 0.393 | |
| P80723 | 9,053.52±2,144.25 | 9,310.73±2,863.67 | 13,318.67±9,043.45 | 21,567.66±11,339.56 | 17,162.37±13,255.24 | 0.103 | |
| P23528 | 738.11±111.92 | 983.84±814.95 | 2,072.42±1,045.52 | 1,236.04±956.59 | 2,031.17±1,730 | 0.134 | |
| **P35222** | **3,096.67±893.18** | **3,177.15±925.4** | **2,188.05±673.87** | **1,844.56±387.51** | **2,604.73±981.01** | **0.009** | 1-3 |
| P55064 | 15,629.24±8,980.83 | 8,386.83±4,887.02 | 8,289.83±3,657.6 | 6,827.04±3,931.72 | 10,559.89±10,306.5 | 0.184 | |
| P04083 | **3,197.25±1,322.34** | **3,029.31±825.01** | **6,718.94±4,762.69** | **12,803.13±9,900.89** | **8,414.14±4,684.5** | **0.046** | 1-3 |
| Q8N9V7 | 25,280.65±13,770.99 | 32,947.31±3,435.2 | 39,202.73±15,405.72 | 51,872.43±31,639.8 | 64,667.44±36,402.65 | 0.112 | |
| Q13315 | 2,910.94±1,476.38 | 3,138.14±1,541.29 | 2,378.32±674.93 | 1,823.75±598.86 | 2,856.84±1,551.16 | 0.173 | |
| Q06830 | 1,288.79±816.54 | 1,105.39±704.42 | 960.99±551.68 | 830.17±178.7 | 1,205.01±485.22 | 0.528 | |
| **Q03135** | **9,418.84±2266.91** | **7,337.52±2,347.32** | **6,528.98±2,741.65** | **4,335.08±1,146.23** | **8,195.92±4,796.14** | **0.021** | 0-3 |
| P60891 | 2,934.62±1,203.47 | 2,239.41±696.17 | 2,143.37±559.68 | 1,618.78±624.01 | 1,675.12±913.62 | 0.054 | |
| Q9Y281 | 9,966.88±2,043.38 | 8,903.64±1,764.81 | 10,871.93±4,370.18 | 7,143.97±2,412.77 | 10,743.89±3,935.25 | 0.129 | |
| P06744 | 2,340.21±765.46 | 2,545.84±1,252.04 | 2,180.35±755.58 | 1,639.56±777.34 | 2,154±845.21 | 0.357 | |
| P36405 | 2,257.79±1,174.66 | 1,765.46±453.44 | 2,088.42±739.08 | 1,942.54±315.34 | 2,193.49±646.19 | 0.758 | |

| | | | | | | | |
|---|---|---|---|---|---|---|---|
| P00918 | 5,990.69±1,123.71 | 4,614.73±1,070.27 | 7,171.48±3,452.93 | 6,856.88±2,429.98 | 6,283.58±828.08 | 0.396 | |
| **P61204** | **4,875.83±2,090.92** | **3,352.33±1,612.17** | **3,162.77±750.15** | **2,191.11±1,068.11** | **3,133.7±1383.35** | **0.027** | 0-3 |
| Q14690 | 7,884.22±3,733.72 | 4,694.77±2,119.32 | 5,302.31±1,954.3 | 4,097.34±798.67 | 5,315.45±2,685.71 | 0.076 | |
| P48163 | 87,595.64±43,559.86 | 11,2562.76±61,987.06 | 107,961.44±41,432.13 | 131,362.39±34,416.33 | 82,529.59±67,211.42 | 0.355 | |
| P04264 | 960.03±346.52 | 1,889.41±2,359.32 | 1,552.86±1,607.66 | 1,137.36±473.05 | 1,076.93±321.65 | 0.738 | |
| O15020 | 348.39±168.68 | 571.43±511.42 | 878.16±103.22 | 1,089.63±1,086.56 | 715.76±451.88 | 0.622 | |
| P18085 | 2,071.14±772.94 | 1,774.6±919.72 | 1,464.56±630.58 | 1,012.96±331.42 | 1,852.63±955.69 | 0.063 | |
| P11844 | 33,683.77±21,530.96 | 26,849.8±15,447.64 | 39,048.13±23,905.36 | 46,815.23±21,262.16 | 23,596.92±28,803.78 | 0.333 | |
| P07205 | 2,311.28±610.43 | 2,964.92±1,299.7 | 3,279.86±1,963.3 | 4,565.36±2,018.76 | 2,902.07±1,915.43 | 0.202 | |
| **P50991** | **6,488.45±530.23** | **5,205.72±1,625.54** | **5,116.36±1,070.03** | **3,499±1,584.4** | **4,199.88±1,540.79** | **0.009** | 0-3 |
| O75828 | 48,421.13±8,628.86 | 52,795.09±15,173.55 | 78,979.91±26,799.18 | 71251.28±21,678.51 | 89,561.21±36,807.84 | 0.062 | |
| **P26038** | **5,244.52±2,261.7** | **3,372.54±985.56** | **3,353.61±1,231.21** | **2,332.94±877.87** | **3,377.1±440.11** | **0.007** | 0-3 |
| Q99497 | 55,792.31±37,911.67 | 55,703.9±18,497.06 | 74,043.57±31,067.44 | 60,607.88±24,673.79 | 87,659.76±30,881.11 | 0.304 | |
| P30086 | 8,180.14±3,485.99 | 8,994.32±2,355.74 | 9,855.91±3,781.66 | 11,467.58±3,308.09 | 11,628.09±1,492.22 | 0.307 | |
| P32754 | 5,057.63±2,946.66 | 9,256.77±4,293.52 | 8,576.07±3,835.01 | 7,995.48±1,838.88 | 10,884.45±6,013.41 | 0.245 | |
| P11171 | **14,455.91±2,956.05** | **1,1865.29±1,490.13** | **11,804.21±3,088.28** | **9,375.15±1,944.55** | **12,019.06±3,192.45** | **0.031** | 0-3 |
| P38606 | 7,631.75±2,852.19 | 6,435.9±1,932.56 | 5,979.4±2,054.28 | 4,658.75±891.66 | 5,999.35±1,274.12 | 0.081 | |
| P11413 | 9,622.4±2,502.36 | 8,962.8±1,467.48 | 8,730.64±1,992.3 | 7,205.2±3,069.64 | 9,336.97±2,012.29 | 0.344 | |
| **Q6UWM7** | **27,488.42±6,800.96** | **21,741.2±4,591.98** | **15,619.62±4,212.23** | **11,413.22±3,485.73** | **14,832.32±3,233.34** | **<0.001** | 0-2, 0-3, 0-4, 1-3 |
| P07437 | **2,071.9±414.59** | **1,409.59±848.23** | **1,299.13±526.36** | **846.46±485.44** | **1,476.56±873.65** | **0.034** | 0-3 |
| P22061 | 19,340.94±7,647.02 | 13,778.84±4342.1 | 15,682.39±7,750.7 | 11,334.28±3,196.95 | 1,6915±5,625.1 | 0.168 | |
| P18669 | 42,59.31±1,916.96 | 5,429.82±3,842.95 | 4,445.06±3,428.84 | 3,167.35±1,126.56 | 4,884.92±2,788.66 | 0.590 | |
| **P04792** | **105,583.93±25,459.1** | **54,021.16±19,028.13** | **55,740.84±28,222.7** | **42,373.41±17,238.9** | **63,843.43±17,265.62** | **0.001** | 0-1, 0-2, 0-3 |
| Q9NY65 | 3361.71±1055.65 | 3,079.22±494.43 | 4,109.33±1,438.74 | 5,034.3±2,165.7 | 5,006.8±1,513.64 | 0.146 | |
| **P31150** | **45,033.81±17,267.85** | **21,607.92±9,973.56** | **19,905.39±5,870.51** | **18,708.12±6,367.76** | **18,752.13±6,418.12** | **<0.001** | 0-1, 0-2, 0-3, 0-4 |
| **Q14240** | **2,455.95±534.33** | **2,127.75±473.83** | **1,781.72±365.11** | **1,263.61±296.99** | **1,842.8±626.09** | **0.001** | 0-3, 1-3 |
| P04075 | 85,763.56±23,178.3 | 8,4427.74±1,1051.1 | 97,392.52±24,921.02 | 126,006.16±46,654.71 | 126,401.51±44,645.93 | 0.100 | |
| P60842 | **16,690.46±3,952.01** | **12,131.85±2,553.81** | **10,333.05±4,111.04** | **10,241.42±3,331.16** | **9,506.6±1,700.71** | **0.022** | 0-2, 0-3, 0-4 |
| P50395 | 57,462.62±17,888.07 | 33,079.1±23,025.92 | 39,084.86±16,695.4 | 27,821.44±15,334.25 | 31,363.82±12,440.63 | 0.075 | |
| P30041 | **109,960.91±50,389.48** | **76,647±23,825.21** | **59,167.44±15,849.04** | **54,280.04±15,607.38** | **64,430±16,125.04** | **0.005** | 0-2, 0-3 |
| P07316 | **33,496.31±7,338.16** | **37,790.49±4,040.66** | **44,110.22±15,886.91** | **63,734.18±26,733.82** | **74,644.1±22,637.07** | **0.006** | 0-4 |
| P68032 | 4,919.73±4,162.52 | 9,625.17±6,762.69 | 6,450.19±4,678.52 | 10,234.32±4,223.8 | 9524.7±1,259.19 | 0.563 | |
| P08238 | **34,044.43±7,473.8** | **32,885.5±10,755.49** | **42,720.47±14,772.85** | **49,557.28±24,875.94** | **68,983.99±24,748.49** | **0.040** | 1-4 |
| P11216 | 87,780.87±16,501.05 | 96,734.63±14,821.74 | 113,365.74±32,066.84 | 132,802.32±52,626.49 | 154,441.28±68,062.58 | 0.131 | |
| P07315 | **36,1821.98±28,069.97** | **363,890.01±80,650.11** | **592,778.66±212,088.86** | **613,270.31±188,857.04** | **678,300.2±257,196.54** | **0.026** | 0-4, 1-4 |
| P08670 | **107,867.42±42,155.62** | **71,713.9±35,546.06** | **60,359.62±23,734.4** | **40,936.92±20,409.67** | **81,551.51±51,360.25** | **0.018** | 0-3 |
| P07320 | **623,645.93±63,828.83** | **618,976.87±113,355.83** | **910,775.48±290,287.73** | **1,076,544.98±437,566.04** | **1,180,908.83±333,929.44** | **0.023** | 1-4 |
| P04406 | **837,474.77±366,921.69** | **483,003.09±157,667** | **524,538.95±220,555.52** | **383,441.91±178,444.27** | **54,2121.55±11,5742.36** | **0.022** | 0-3 |
| P22914 | **1,313,705.63±214,921.34** | **1,460,229.19±247,885.76** | **2,069,107.57±615,423.61** | **2,663,219.11±1,194,808.49** | **2,685,583.23±802,164.07** | **0.020** | 0-3, 0-4 |
| P53674 | **1,934,017.76±343,098.53** | **2,442,628.18±532,089.55** | **2,856,085.9±635,617.26** | **3,469,388.63±821,945.92** | **3,420,450.6±632,490.04** | **0.003** | 0-3, 0-4 |
| **Q12934** | **1,535,183.91±331,417.11** | **1,326,410.12±330,571.67** | **1,120,305.37±202,272.41** | **877,036.25±204,133.15** | **1,191,278.83±242,500.75** | **0.001** | 0-3, 1-3 |
| **Q13515** | **2,126,436.05±581,628.87** | **1,707,856.47±362,461.42** | **1,474,621.72±264,254.02** | **1,116,858.39±281,702.66** | **1,572,205.5±295,006.32** | **<0.001** | 0-2, 0-3, 1-3 |

**STD:** Standart Deviation. The proteins that vary in expression between transparent lenses and cataract types were shown in bold letters.
The last column, "difference", shows mutually differing groups with statistical significance, represented by the numbers written before the cataract types.

**Supplementary Table S4.** Proteins Classified According to Visual Acuity and Dysfunctional Lens Index

| Visual Acuity (LogMAR) | | | | | | Dysfunctional Lens Index | | | | | |
|---|---|---|---|---|---|---|---|---|---|---|---|
| **Protein** | **r*** | **p#** | **Protein** | **r** | **p** | **Protein** | **r** | **p** | **Protein** | **r** | **p** |
| **P13489** | **-0.414** | **0.017** | **Q14690** | **-0.366** | **0.036** | **P13489** | **0.379** | **0.039** | Q14690 | 0.119 | 0.531 |
| Q9H4G0 | -0.191 | 0.286 | P48163 | 0.325 | 0.065 | Q9H4G0 | 0.091 | 0.634 | P48163 | -0.283 | 0.130 |
| **Q6NZI2** | **-0.413** | **0.017** | P04264 | -0.108 | 0.550 | **Q6NZI2** | **0.391** | **0.033** | P04264 | 0.212 | 0.261 |
| **Q6ZMZ3** | **0.490** | **0.004** | O15020 | 0.294 | 0.096 | **Q6ZMZ3** | **-0.410** | **0.024** | O15020 | 0.101 | 0.596 |
| **Q9NYC9** | **0.508** | **0.003** | **P18085** | **-0.502** | **0.003** | Q9NYC9 | -0.347 | 0.060 | **P18085** | **0.407** | **0.026** |
| **P21281** | **-0.438** | **0.011** | P11844 | 0.201 | 0.262 | P21281 | 0.306 | 0.100 | P11844 | -0.341 | 0.066 |
| **B1AJZ9** | **0.453** | **0.008** | **P07205** | **0.534** | **0.001** | B1AJZ9 | -0.238 | 0.205 | P07205 | -0.143 | 0.452 |
| **P14550** | **0.412** | **0.017** | **P50991** | **-0.600** | **<0.001** | P14550 | -0.332 | 0.073 | **P50991** | **0.438** | **0.015** |
| **Q13449** | **-0.539** | **0.001** | O75828 | 0.218 | 0.224 | **Q13449** | **0.453** | **0.012** | O75828 | -0.150 | 0.430 |
| **Q9BYB0** | **-0.527** | **0.002** | P26038 | **-0.459** | **0.007** | Q9BYB0 | 0.280 | 0.134 | **P26038** | **0.468** | **0.009** |
| **P00387** | **-0.389** | **0.025** | **Q99497** | 0.027 | 0.882 | P00387 | 0.259 | 0.168 | Q99497 | 0.003 | 0.989 |
| **O76054** | **-0.608** | **<0.001** | **P30086** | **0.381** | **0.029** | **O76054** | **0.565** | **0.001** | P30086 | -0.287 | 0.124 |
| **P34913** | **-0.439** | **0.011** | P32754 | 0.131 | 0.467 | **P34913** | **0.481** | **0.007** | P32754 | 0.228 | 0.225 |
| **P22748** | **-0.370** | **0.034** | **P11171** | **-0.408** | **0.018** | P22748 | 0.336 | 0.070 | **P11171** | **0.459** | **0.011** |
| P13645 | -0.280 | 0.115 | **P38606** | **-0.400** | **0.021** | **P13645** | **0.420** | **0.021** | **P38606** | **0.459** | **0.011** |
| P63000 | -0.008 | 0.964 | **P11413** | **-0.347** | **0.048** | P63000 | 0.010 | 0.958 | P11413 | 0.351 | 0.057 |
| Q99832 | -0.184 | 0.305 | **Q6UWM7** | **-0.519** | **0.002** | Q99832 | -0.137 | 0.470 | **Q6UWM7** | **0.475** | **0.008** |
| **P27361** | **-0.366** | **0.036** | **P07437** | **-0.483** | **0.004** | P27361 | 0.135 | 0.477 | **P07437** | **0.382** | **0.037** |
| O15498 | -0.342 | 0.051 | **P18669** | **-0.347** | **0.048** | O15498 | 0.297 | 0.111 | P18669 | 0.269 | 0.151 |
| **Q9ULX7** | **-0.427** | **0.013** | **P04792** | **-0.482** | **0.005** | **Q9ULX7** | **0.372** | **0.043** | P04792 | 0.324 | 0.081 |
| **Q96M86** | **0.575** | **<0.001** | **Q9NY65** | **0.537** | **0.001** | **Q96M86** | **-0.398** | **0.029** | Q9NY65 | -0.272 | 0.146 |
| P07196 | -0.169 | 0.346 | P31150 | -0.317 | 0.073 | P07196 | 0.193 | 0.307 | P31150 | 0.246 | 0.189 |
| Q9H1H9 | -0.213 | 0.234 | **Q14240** | **-0.454** | **0.008** | Q9H1H9 | 0.149 | 0.431 | **Q14240** | **0.486** | **0.007** |
| Q9Y6H8 | -0.179 | 0.318 | **P04075** | **0.640** | **<0.001** | Q9Y6H8 | 0.106 | 0.576 | P04075 | -0.318 | 0.087 |
| P53672 | -0.289 | 0.103 | P60842 | -0.336 | 0.056 | P53672 | 0.300 | 0.107 | P60842 | 0.290 | 0.120 |
| **P80723** | **0.728** | **<0.001** | **P50395** | **-0.409** | **0.018** | **P80723** | **-0.465** | **0.010** | P50395 | 0.333 | 0.072 |
| P23528 | -0.051 | 0.777 | P30041 | -0.291 | 0.100 | P23528 | -0.062 | 0.745 | P30041 | 0.315 | 0.090 |
| **P35222** | **-0.390** | **0.025** | **P07316** | **0.680** | **<0.001** | P35222 | 0.293 | 0.116 | **P07316** | **-0.424** | **0.020** |
| P55064 | 0.037 | 0.839 | **P68032** | **0.409** | **0.018** | P55064 | -0.055 | 0.772 | P68032 | -0.248 | 0.187 |
| **P04083** | **0.642** | **<0.001** | P08238 | 0.286 | 0.107 | **P04083** | **-0.425** | **0.019** | P08238 | -0.304 | 0.102 |
| **Q8N9V7** | **0.482** | **0.005** | **P11216** | **0.514** | **0.002** | Q8N9V7 | -0.329 | 0.076 | P11216 | -0.349 | 0.058 |
| **Q13315** | **-0.360** | **0.040** | **P07315** | **0.529** | **0.002** | Q13315 | 0.375 | 0.041 | P07315 | **-0.464** | **0.010** |
| Q06830 | -0.139 | 0.440 | **P08670** | **-0.471** | **0.006** | Q06830 | 0.193 | 0.306 | P08670 | 0.228 | 0.225 |
| Q03135 | -0.129 | 0.474 | **P07320** | **0.632** | **<0.001** | Q03135 | 0.165 | 0.384 | **P07320** | **-0.452** | **0.012** |
| Q9Y281 | -0.271 | 0.127 | **P22914** | **0.661** | **<0.001** | Q9Y281 | 0.154 | 0.416 | **P22914** | **-0.455** | **0.012** |
| P06744 | -0.244 | 0.171 | **P53674** | **0.624** | **<0.001** | P06744 | 0.343 | 0.064 | **P53674** | **-0.458** | **0.011** |
| P36405 | -0.201 | 0.262 | **Q12934** | **-0.475** | **0.005** | P36405 | 0.302 | 0.105 | Q12934 | 0.313 | 0.092 |
| P00918 | 0.081 | 0.656 | **Q13515** | **-0.490** | **0.004** | P00918 | -0.299 | 0.108 | **Q13515** | **0.369** | **0.045** |
| **P61204** | **-0.528** | **0.002** | **P60891** | **-0.620** | **<0.001** | **P61204** | **0.523** | **0.003** | **P60891** | **0.529** | **0.003** |

*r= Correlation coefficient. #p: Statistical significance
